# Perceptions of undergraduate midwifery students regarding academic self-efficacy at the selected Nursing Education Institutions in Gauteng province

**DOI:** 10.1016/j.mex.2026.103954

**Published:** 2026-05-12

**Authors:** Nkhensani Florence Mabunda, Dorica Motaung, Yvonne Rambuwani

**Affiliations:** Sefako Makgatho Health Sciences University, Molotlegi Street, Ga-Rankuwa, Pretoria, Gauteng. P.O Box 60, Medunsa 0204, South Africa

**Keywords:** Academic self-efficacy, Descriptive, Explorative, Qualitative, Midwifery students, Perceptions

## Abstract

Understanding how undergraduate midwifery students view their academic self-efficacy is crucial for gaining a deeper insight into their learning experiences, levels of confidence, and preparedness to perform competently in clinical settings. Academic self-efficacy not only affects students’ motivation and academic performance but also impacts how they manage academic pressures and the emotional demands of midwifery training. By identifying the factors that influence students’ perceptions and by implementing targeted, evidence-based strategies to enhance both academic and psychological support, Nursing Education Institutions can improve students’ learning outcomes and overall well-being. This qualitative research study aims to gain insights into undergraduate midwives' perceptions of their academic and psychological well-being at the selected Nursing Education Institutions in Gauteng province. The study is grounded in Bandura’s Social Cognitive Theory, which offers a comprehensive lens for examining how personal beliefs, environmental conditions, and behavioural factors interact to shape students’ learning experiences and coping strategies. The results will help students understand factors influencing their academic journey, develop strategies to overcome challenges and inform policy decisions related to midwifery education and training, and guide institutional policies aimed at strengthening midwifery education and student support systems.

## Graphical abstract

**Figure:**
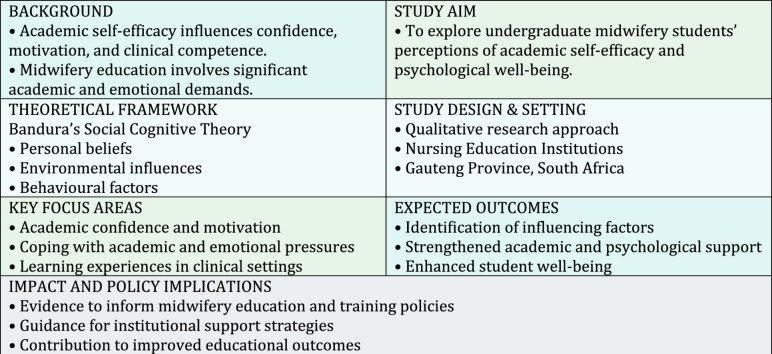

Specifications table

**Table:** 

**Subject area**	Psychology
**More specific subject area**	Nursing Education
**Name of your protocol**	Perceptions of undergraduate midwifery students regarding academic self-efficacy at the selected Nursing Education Institutions in Gauteng province
**Reagents/tools**	Not applicable
**Experimental design**	Not applicable
**Trial registration**	SMUREC/H/450/2024:PG
**Ethics**	Written informed consent from undergraduate midwifery nursing students will be obtained before collecting any data.
**Value of the Protocol**	• Provides a clearer understanding of the factors that shape midwifery students’ academic self-efficacy and psychological well-being, thereby supporting improved learning outcomes and clinical readiness. • Offers guidance to Nursing Education Institutions in designing evidence-based academic, psychosocial, and mentorship strategies aimed at boosting student achievement and retention. • Adds to the body of midwifery education research by utilising Social Cognitive Theory within a South African context, informing policy formulation and reinforcing training programmes.

## Background

Academic self-efficacy is a student's belief in their ability to succeed in academic tasks. This belief is especially important in demanding fields like midwifery [1]. As noted in the literature, high self-efficacy improves learning outcomes, motivation, resilience, and the mastery of both theoretical and practical skills, thereby preparing students for professional practice. Low self-efficacy, by contrast, leads to avoidance of new skills and help-seeking, causing frustration, anxiety, and hindering students’ progress and readiness to provide quality care [2]. Even though individuals with higher self-efficacy set more ambitious goals, take on greater challenges, perform academic tasks more effectively, and use a wider range of learning strategies [3]. Students’ perceptions of their learning experiences can strengthen or weaken their academic self-efficacy.

According to the International Code of Ethics for Midwives, midwives must use up-to-date, evidence-based knowledge to remain competent and provide safe care in all settings [4], which presupposes adequate academic self-efficacy. Yet, the International Council of Nurses reports a severe nursing workforce shortage. Nursing and midwifery students face stress, burnout, and intentions to leave the profession, similar to registered midwives [5]. Declining student numbers threaten the future of midwifery and worsen the global nursing. The 2021 State of the World’s Midwifery Report identified a global shortage of 900,000 midwives, contributing to major maternal health problems, including 810 maternal deaths daily, a stillbirth every 16 s, and 2.4 million newborn deaths each year [6].

In South Africa, the 2022 "Saving Mothers Report" recorded 1062 maternal deaths and a Maternal Mortality Ratio of 109.6 per 100,000 live births. Over half (52.6%) of maternal deaths in 2021 were preventable, with low staffing and inadequate skills responsible for 7% and 9.8% of deaths, respectively [7,8]. These findings underscore the need to invest in midwifery education. In South Africa, midwifery education is delivered through the Advanced Diploma in Midwifery (R.1497). The inaugural R.1497 group commenced in January 2024 to prepare competent, independent midwives [9]. These students, however, began their training without prior orientation to maternity units. New teaching strategies, an updated curriculum, and greater use of technology may impede learning and diminish motivation, engagement, performance, and academic self-belief. Consequently, students’ confidence and preparedness for practice may be compromised. While undergraduate midwifery students need robust academic self-efficacy, high workloads, challenging clinical settings, and performance expectations can heighten stress and contribute to burnout. There is limited evidence on how these curriculum reforms affect academic self-efficacy in public NEIs, especially within Gauteng Province.

The study extends beyond academic self-efficacy and additionally incorporates psychological well-being as a complementary construct. Academic self-efficacy is defined as students’ confidence in their capacity to successfully carry out academic and clinical activities, whereas psychological well-being concerns their emotional and mental state throughout training. These two constructs are interconnected: higher levels of self-efficacy are linked to lower stress and better coping strategies, which in turn promote well-being, while compromised psychological well-being can erode confidence and hinder performance (Albert Bandura; World Health Organization, 2022; Sarah Pressman et al., 2023). Accordingly, the study investigates both constructs and their interplay, maintaining consistency across all sections.

This context prompted the researcher to investigate midwifery students’ lack of academic motivation and self-doubt in acquiring midwifery knowledge and clinical skills, which led to poor performance. Exploring undergraduate midwifery students’ perceptions of their academic self-efficacy can provide insight into their learning experiences and readiness for clinical practice. By identifying factors influencing these perceptions, Nursing Education Institutions can design targeted interventions to improve students’ learning outcomes and well-being.

## Description of protocol

### Background information

Academic self-efficacy refers to students’ beliefs about their capability to succeed in academic tasks. This belief is particularly crucial in rigorous disciplines such as midwifery. Examining undergraduate midwifery students’ perceptions of their own academic self-efficacy can shed light on their learning experiences and their preparedness for clinical practice. By determining the factors that shape these perceptions, Nursing Education Institutions can develop targeted strategies to enhance students’ learning outcomes and overall well-being.

### Study rationale

New midwifery programmes in South Africa, such as the Advanced Diploma in Midwifery (R.1497), have reshaped education in NEIs. Limited orientation, unfamiliar teaching methods, and growing technological demands may affect students’ academic self-efficacy and psychological well-being. Academic self-efficacy is vital for motivation, learning, clinical performance, and readiness for practice, while low self-efficacy raises stress, anxiety, and burnout, harming academic performance, programme completion, and patient care. However, little is known about how undergraduate midwifery students in public NEIs in Gauteng perceive their academic self-efficacy in this changing context. This study addresses this gap by exploring their experiences, challenges, and support needs.

### Study purpose and objectives

The study seeks to explore how undergraduate midwifery students at the selected NEIs in Gauteng Province perceive their own academic self-efficacy.

The objective of the study is to explore and describe the views of undergraduate midwifery students on academic self-efficacy at the selected NEIsin Gauteng Province

### Theoretical framework

This study is based on Bandura's Social Cognitive Theory, which frames how undergraduate midwifery students in Gauteng NEIs perceive academic self-efficacy. Bandura [10] argues that self-efficacy is central to behaviour, motivation, and emotional well-being and is a key concept in psychology and education. It supports the development of clinical skills, critical thinking, and academic achievement. The theory guides this investigation into how students’ beliefs about their academic abilities and coping skills affect their performance and persistence, informing interventions to enhance academic success. It also enables educators and researchers to understand how self-efficacy and learning strategies shape students’ academic experiences and professional growth [11]. According to Schunk and DiBenedetto [12], Social Cognitive Theory proposes a reciprocal relationship between personal factors (e.g., self-efficacy), environmental factors (e.g., learning environment), and behaviour (e.g., academic performance), which continuously influence one another.

### Definition of concepts

Perceptions: refers to the process by which we select, organize, and interpret information inputs to create a meaningful picture of the world [13]. In this study, perception refers to the way student midwives interpret, understand, and experience the world around them.

Academic self-efficacy: refers to one’s belief about his/her capabilities to accomplish specific tasks [14]. In this study, academic self-efficacy refers to a student's belief in their ability to succeed in academic tasks or achieve a particular goal.

Undergraduate Midwifery Students: defined as a person enrolled in a formal midwifery program in a Higher Education Institution who is Registered with the SANC as a student in terms of the Nursing Act 33 of 2005 [15] In this study undergraduate midwifery student is a person enrolled for the Advanced Diploma in Midwifery programme in one of the accredited Gauteng Colleges of Nursing and registered with the SANC as a learner.

## Methodology

In this study, the methodology will include qualitative research design, methods, the data collection procedures, and the analytical techniques used to address the research question.

### Design

Qualitative research is the investigation of phenomena through the collection of rich narrative materials using a flexible research design [16]. In this study, the researcher will use a qualitative research approach and an explorative descriptive design to explore students' perceptions regarding academic self-efficacy. These approaches will allow the researchers to acquire a comprehensive understanding of participants' viewpoints on their programs, examine their challenges, and determine ways to overcome them [17]. Therefore, a qualitative, exploratory, and descriptive research design is deemed appropriate for this study, as it is oriented toward the development of an in-depth understanding of the phenomenon under investigation and enables the researcher to obtain rich, refined, and contextually detailed data. In addition, this study is grounded in Bandura’s Social Cognitive Theory [18], which asserts that human functioning emerges from the reciprocal interaction of personal factors, behaviour, and environmental conditions. In this context, personal factors comprise midwifery students’ academic self-efficacy, motivation, and emotional reactions to learning. Behavioural factors involve students’ study habits, participation in academic tasks, and tendencies to seek help. Environmental factors include institutional support, instructional approaches, clinical training settings, and the influence of peers. The theory further guides an examination of mastery experiences, observational learning, verbal encouragement, and emotional conditions as primary contributors to students’ academic self-efficacy [19]. These constructs informed the development of the interview protocol and the thematic analysis framework.

### Study setting

The study will be conducted on selected Gauteng College of Nursing (GCON) campuses in Gauteng Province that offer the Advanced Diploma in Midwifery program R.1497. GCON is a newly established institution formed by merging all provincial nursing colleges into a single college with six campuses, each with 50 enrolled students. Gauteng’s heterogeneous, urban population provides access to diverse NEIs offering midwifery programs that train future midwives in the theoretical and practical skills needed for safe, effective practice. A multi-site approach will enable the collection of varied perspectives, enhancing the quality and scope of the qualitative data.

### Study population and sampling

The target population will include undergraduate midwifery students enrolled in the Advanced Diploma in Midwifery program who have already completed both the clinical and theoretical components of the curriculum. In this study, non-probability purposive sampling will be used to ensure that the selected students meet specific preselected criteria to select participants who can offer in-depth and relevant information [20]. The accessible population for this study will include Undergraduate Midwifery Students enrolled for the Advanced Diploma in Midwifery (R.1497) at the selected NEIs in Gauteng. The researcher will work in collaboration with the management and academic coordinators of the selected Nursing Education Institutions (NEIs) in Gauteng to obtain permission and identify suitable participants. Thereafter, the researcher will meet prospective participants during scheduled academic sessions to explain the study in detail and invite them to take part voluntarily. An information sheet describing the purpose of the study, the procedures involved, as well as possible risks and anticipated benefits, will be provided, and written informed consent will be secured before any participation takes place. Participants will be informed that their involvement is entirely voluntary and that they may discontinue their participation at any time without any academic or personal repercussions.

The term “sample size” denotes the total number of individuals involved in the research. In qualitative studies, sample size is determined by informational needs, often described as data saturation [21]. Therefore, the researcher will seek to recruit 10–15 participants, with the actual number finalized once no new themes or perspectives arise and the data begin to repeat, signaling that further data collection is unnecessary and that redundancy has been achieved. This study will include all undergraduate midwifery students who are presently registered in the Advanced Diploma in Midwifery program (R.1497) and who consent to take part in the research. Undergraduate students enrolled in the Advanced Diploma in Midwifery (R.1497) program who are ill or on leave will be excluded.

### Data collection

This study will use semi-structured, face-to-face interviews with open-ended questions [22]. Participants will be undergraduate midwifery students enrolled in the R.1497 program at the three GCON campuses. Data will be collected after approval from the Gauteng Department of Health and permission from campus heads. The researcher will travel to the institutions, obtain written informed consent, and brief participants on the study’s purpose and confidentiality before conducting interviews. Interviews will be conducted in English and will be audio recorded with permission from the participants. One central question: “How would you describe your confidence in your capacity to succeed in both the academic and clinical components of your midwifery training?”

Although the primary research question is grounded in Bandura’s Social Cognitive Theory, directing data collection toward the interplay of personal, behavioural, and environmental factors that shape academic self-efficacy [22]. It examines students’ beliefs, study practices, and learning environments, with particular attention to mastery experiences, modelling, and feedback. In doing so, it provides a theoretically informed perspective on how self-efficacy is formed and sustained within nursing education contexts.

Probing questions will be asked in interviews to draw out more detailed information, clarify unclear responses, reveal context, reduce assumptions, and enhance overall understanding [23], depending on the response from participants. In addition, the researcher will also use communication skills to ensure rich, thick descriptions are obtained. Each interview will last 30–60 min, depending on the conversation and participants’ willingness to share their perceptions of academic self-efficacy and psychological well-being. To minimise interruptions, interviews will be conducted in a quiet, private setting, with mobile phones switched off, advance instructions provided, and the importance of uninterrupted time communicated. With participants’ written consent, interviews will be audio-recorded, and recordings will be stored securely and confidentially.

A pre-test will be conducted to refine the interview guide, assess feasibility, and identify methodological issues [24]. The interview guide will be tested on five undergraduate midwifery students from NEIs who meet the inclusion criteria but will not be part of the main study.

### Data analysis

In this study, qualitative data will be analysed using a thematic analysis approach, guided by Xu and Zammit [25] and Braun and Clarke [26]. Audio recordings will be transcribed verbatim and read multiple times to ensure thorough familiarisation. Familiarisation with the data: The researcher will transcribe all interviews verbatim and read through the transcripts multiple times while simultaneously listening to the audio recordings to ensure accuracy. Initial notes will focus on how students talk about their confidence, the challenges they face, the strategies they adopt to manage these challenges, and their experiences in both academic and clinical settings.

Generating initial codes: All relevant segments of the data will be systematically coded. Possible codes include aspects such as confidence in clinical skills, fear of failure, academic demands, support from lecturers, and working collaboratively with peers. Coding will be largely data-driven to accurately represent participants’ reported experiences.

Searching for themes: The codes will then be grouped into broader themes that represent recurring patterns related to students’ academic self-efficacy. Emerging themes will be shaped by ideas that consistently appear across multiple participants.

Reviewing themes: These preliminary themes will be critically reviewed and refined by checking them against the coded data extracts and the entire dataset. Overlapping themes will be merged, and any unclear or weak themes will be modified or removed to preserve coherence and clarity.

Defining and naming themes: The final set of themes will be clearly defined and assigned descriptive labels that convey their importance in relation to academic self-efficacy within midwifery education.

Producing the report: The findings will be presented in a narrative account, supported by direct quotations from participants. The discussion will link these results to the existing literature on self-efficacy and midwifery education.

Additionally, data will be managed by removing identifying information, storing it securely, and protecting it with passwords. Audio files, transcripts, and field notes will be saved on encrypted devices, with backup copies on secure external drives. Any hard-copy materials will be kept in locked storage. All data will be retained for five years and then securely destroyed, in line with ethical guidelines and institutional policies.

### Trustworthiness

To uphold trustworthiness in this, Lincoln and Guba's [27] criteria [28] of credibility, transferability, dependability, and confirmability will be employed. Credibility will be promoted through sustained engagement with participants, member validation of findings, and, where possible, triangulation of data sources. Transferability will be supported by providing thick, detailed descriptions of the study setting, participants, and the learning environment within the selected Nursing Education Institutions. Dependability will be ensured by documenting a transparent audit trail of all methodological decisions, including data collection processes and steps in the thematic analysis. Confirmability will be reinforced through reflexive journaling and the maintenance of an audit trail, ensuring that the findings reflect participants’ accounts rather than the researcher’s preconceptions.

In addition, potential researcher bias is recognised, particularly because the researcher may be familiar with nursing education settings and hold assumptions about academic self-efficacy. Such factors could shape how the data are interpreted or how questions are posed during interviews. To reduce bias, the researcher will engage in reflexivity, regularly examining personal beliefs and how they may affect the research process [29]. In addition, an independent peer reviewer or supervisor will be invited to review the coding process and the development of themes, helping to ensure that the results are impartial and accurately reflect participants’ views. The use of a semi-structured interview guide based on Bandura’s Social Cognitive Theory will further promote consistency and limit the risk of leading questions, in line with current recommendations for qualitative research design.

### Ethical consideration

The following ethical considerations will be considered in the study.

### Ethical clearance

The study proposal and tools were submitted to the Sefako Makgatho Health Science University Research Ethics Committee for ethical clearance SMUREC/H/450/2024:PG.

### Ethics statements

Ethical clearance will be obtained from Sefako Makgatho Health Science University Research Ethics (SMUREC) before the commencement of the research project. Permission to conduct the study from the Gauteng Department of Health and from the Campus Heads of the selected campuses of the NEIs in Gauteng Province. Written informed consent from participants will be sought before data collection [30]. The following ethical principles will be adhered to

### Anonymity

Names and other identifying details of participants will be safeguarded through the use of pseudonyms. The researcher will ensure that no data can be traced back to any individual participant. Care will be taken in this process, and participants will be labeled with numerical codes or alphabetical characters.

### Confidentiality privacy

All personal information obtained from participants will be treated as strictly confidential. Data, including audio recordings and field notes, will be stored in a locked, ventilated room, accessible only to the principal investigator, supervisor, and co-supervisor. Any facilitators involved will sign confidentiality and privacy agreements confirming their obligation not to disclose study content or identifying participant information.

### Beneficence

This principle concerns freedom from harmful treatment. In this study, the researcher will minimise harm to participants while maximising potential benefits. This will be achieved by consistently respecting human dignity, justice, objectivity, and integrity throughout the research process.

### Respect for human dignity

This principle affirms that every participant must be treated as a human being to honor their inherent dignity. Following these principles ensures that researchers and participants uphold the rights to human dignity, full and honest disclosure, and self-determination.

### Justice

The ethical principle of justice will guide this study by ensuring fairness in participant selection, treatment, and data management. Recruitment will be transparent and equitable, avoiding discrimination or exploitation, with particular attention to vulnerable individuals and groups. All eligible participants will have an equal chance to enroll, and no subgroup will face a disproportionate burden or exclusion without a clear scientific justification.

### Objectivity and integrity in research

The researcher will maintain objectivity and integrity throughout the research. Any limitations affecting the findings’ validity will be fully disclosed, and the researcher will not alter data in ways that could influence the study’s results.

### Dissemination and implementation of results

In this research protocol, the dissemination of results will center on the primary research argument derived from the study’s findings. These results will be shared to contribute to and broaden the existing scientific literature. The research report will be submitted to a peer-reviewed journal and presented at academic conferences. In addition, copies of the report will be provided to the Gauteng College of Nursing campuses, and participants will be informed of the research outcomes.

## Protocol validation

To validate the study protocol, a pilot study will be conducted with five undergraduate midwifery students at a selected Nursing Education Institution in Gauteng Province. The semi-structured interview guide will be assessed for clarity, relevance, and appropriate length. All participants completed the interviews within 30–40 min, and no items were considered unclear, supporting its face validity. Feedback from academic staff and subject experts confirmed that the content is suitable, thereby reinforcing content validity. Participants are expected to offer rich, meaningful perspectives on academic self-efficacy and psychological well-being, suggesting that the protocol can successfully elicit the intended information. The pilot also verified that recruitment, data collection, and transcription processes are feasible, demonstrating that the protocol is both practical and ethically appropriate for the main study.

## Limitations

This study is confined to undergraduate midwifery students registered at selected Nursing Education Institutions in the Gauteng Province. It specifically aims to investigate students’ perceptions of their academic self-efficacy and psychological well-being and does not include other nursing disciplines or institutions outside this province. The research uses a qualitative design informed by Bandura’s Social Cognitive Theory and thus captures the subjective experiences and contextual influences described by participants during the data collection period. The focus is restricted to factors shaping learning experiences, confidence, coping mechanisms, and support requirements within the selected institutions, and there is no intention to generalise the results beyond this clearly defined population and context.

## CRediT author statement

Ms Dorica Motaung: Conceptualization, protocol design and development, writing original draft, editing, and revisions. Dr Nkhensani Florence Mabunda: Main research supervisor, Conceptualization, Protocol design and development supervision, guidance, feedback, editing, and revisions. Ms Yvonne Rambuwani: Co-supervisor, Writing review & editing.

## Declaration of competing interest

The authors declare that they have no known competing financial interests or personal relationships that could have appeared to influence the work reported in this paper.

## Data Availability

No data was used for the research described in the article.

## Appendix Supplementary materials

Image, application 1
