# Supplementary material for: Perceptions of undergraduate midwifery students regarding academic self-efficacy at the selected Nursing Education Institutions in Gauteng province
Source: MethodsX. 2026 May 12;16:103954. doi: 10.1016/j.mex.2026.103954 (PMC13197691; doi:10.1016/j.mex.2026.103954)
Supplement: Supplementary file 1 [file mmc1.pdf]

SEFAKO MAKGATHO HEALTH SCIENCES UNIVERSITY

Research & Innovation

Sefako Makgatho University Research Ethics Committee (SMUREC)

CLEARANCE CERTIFICATE - NEW APPLICATION

21 November 2024
Ms DR Motaung
Nursing Science
MEDUNSA
0204

Dear Ms Motaung

MEETING: 10/2024
PROJECT ID: 2450
ETHICS REFERENCE NO: **SMUREC/H/450/2024:PG**

The new Application received was reviewed by members of Sefako Makgatho University Research Ethics Committee on **21 November 2024.**

**Title:**	Perceptions of undergraduate midwifery students regarding academic self-efficacy at a selected Nursing Education Institution in Gauteng province
**Researcher:**	Ms DR Motaung
**Supervisor:**	Dr NF Mabunda
**Co-Supervisor:**	Ms MY Rambuwani
**Department:**	Nursing Science
**School:**	Health Care Sciences
**Type of Study:**	Postgraduate Research
**Degree:**	MCur

Please note the following information about your approved research protocol:

Approval Period: **21 November 2024 - 21 November 2025**

After Ethical Review: Kindly remember to use your protocol number **(SMUREC/H/450/2024:PG)** on any documents or correspondence concerning your research protocol with the REC. The REC has the prerogative and authority to ask further questions, seek additional information, require further modification, or monitor the conduct of your research and the consent process. A template of the progress report is obtainable from the Research Office and is due on an annual basis for your study irrespective of the approval period. Please note that a number of projects may be selected randomly for an external audit every year. Translation of the consent document in the language applicable to the study participants' should be submitted if required. Facility approval must be obtained, and a copy of the letter be submitted to the Committee before commencement of the study.

International Organisation (IORG0008691), **Institutional Review Board** (IRB000010386) Expiry date: 1 8 December 2027, **Federal Wide Assurance** (FWA000023943) Expiry date: 03 November 2026 and **NHREC No: REC 210408-003**

Sincerely

PROF C BAKER

CHAIRPERSON
